# Early and late outcomes of video thoracoscopic versus open approach for bronchiectasis lung resections

**DOI:** 10.1590/0100-6991e-20243748-en

**Published:** 2024-08-22

**Authors:** ANTERO GOMES, LUCAS CASTRO DE OLIVEIRA, FLÁVIO MENDES ALVES, LEONARDO FREIRE ALVES NOGUEIRA, VANESSA FERNANDES OLIVEIRA, ISRAEL LOPES MEDEIROS, ALESSANDRO WASUM MARIANI

**Affiliations:** 1 - Universidade Federal do Ceará, Cirurgia Torácica - Fortaleza - CE - Brasil; 2 - Hospital De Messejana Dr. Carlos Alberto Studart Gomes, Cirurgia Torácica - Fortaleza - CE - Brasil; 3 - Universidade de Sao Paulo (HCFMUSP), Cirurgia Torácica - São Paulo - SP - Brasil

**Keywords:** Surgery, Video-Assisted, Thoracic Surgery, Video-Assisted, Bronchiectasis, Cirurgia Vídeoassistida, Cirurgia Torácica Vídeoassistida, Bronquiectasia

## Abstract

**Objectives:**

To evaluate the early and late results of surgical treatment of patients with bronchiectasis, comparing the Video-Assisted Thoracic Surgery (VATS) vs. the open thoracotomy (OT).

**Methods:**

Observational retrospective study of patients who underwent surgery for bronchiectasis. Patients were divided into two groups according to surgical access OT/VATS. Variables collected included gender, age, preoperative symptoms, etiology, segments involved, FVC and FEV1, type of surgical resection, complications, mortality, and length of hospital stay. Late surgical results were classified as excellent, complete remission of symptoms; good, significative improvement; and poor, little/no improvement.

**Results:**

108 surgical resections (103 patients). OT group 54 patients (52.4%) vs. VATS 49 (47.6%). A high percentage of complications was observed, but no difference between the OT (29.6%) and VATS (24.5%) groups was found. Post-operative hospital stay was shorter in the VATS group (5.4 days) vs. the OT group (8.7 days (p=0.029). 75% of the patients had a late follow-up; the results were considered excellent in 71.4%, good in 26%, and poor in 2.6%. Regarding bronchiectasis distribution, an excellent percentage was obtained at 82.1% in patients with localized bronchiectasis and 47.5% with non-localized bronchiectasis, p=0.003.

**Conclusions:**

VATS leads to similar results regarding morbidity, compared to OT. However, VATS was related to shorter hospital stays, reflecting the early recovery. Late results were excellent in most patients, being better in patients with localized bronchiectasis. VATS should be considered a preferable approach for bronchiectasis lung resection whenever possible.

## RESUMO

**Objetivos:**

Avaliar os resultados precoces e tardios do tratamento cirúrgico de pacientes com bronquiectasias, comparando a Cirurgia Torácica Videoassistida (VATS) versus a toracotomia (TO).

**Métodos:**

Estudo retrospectivo observacional de pacientes submetidos à cirurgia de bronquiectasias. Os pacientes foram divididos em dois grupos de acordo com o acesso cirúrgico TO/VATS. As variáveis coletadas incluíram sexo, idade, sintomas pré-operatórios, etiologia, segmentos envolvidos, CVF e VEF1, tipo de ressecção cirúrgica, complicações, mortalidade e tempo de internação. Os resultados cirúrgicos tardios foram classificados como excelentes, remissão completa dos sintomas; bom, melhora significativa; e pobre, pouca/nenhuma melhoria.

**Resultados:**

108 ressecções cirúrgicas (103 pacientes). Grupo TO 54 pacientes (52,4%) vs. VATS 49 (47,6%). Observou-se alto percentual de complicações, mas não foi encontrada diferença entre os grupos TO (29,6%) e VATS (24,5%). O tempo de internação pós-operatório foi menor no grupo VATS (5,4 dias) versus o grupo TO (8,7 dias) (p=0,029). 75% dos pacientes tiveram seguimento tardio; os resultados foram considerados excelentes em 71,4%, bom em 26% e ruim em 2,6%. Quanto à distribuição das bronquiectasias, o percentual excelente foi de 82,1% em pacientes com bronquiectasias localizadas e 47,5% com bronquiectasias não localizadas, p=0,003.

**Conclusões:**

VATS mostrou resultados semelhantes em relação à morbidade, comparado ao TO. No entanto, a VATS foi relacionada a internações hospitalares mais curtas, refletindo a recuperação precoce. Os resultados tardios foram excelentes na maioria dos pacientes, sendo melhores em pacientes com bronquiectasias localizadas. A VATS deve ser considerada uma abordagem preferível para a ressecção pulmonar de bronquiectasias sempre que possível.

## INTRODUCTION

Initially described by Laennec in the early 19^th^ century^1^ bronchiectasis consists of irreversible dilatation and distortion of the bronchial tree caused by repeated cycles of infection, inflammation, and tissue repair. The classic symptoms include repetitive infections, recurrent hemoptysis, and chronic cough with mucopurulent sputum, alternating with asymptomatic periods or productive cough^2^. It constitutes a disabling disease, which, in addition to damaging the patient’s quality of life, causes a significant financial impact on the public health system^3^. By the end of the 20^th^ century, advances in antibiotic therapy associated with the popularization of vaccination in children contributed to a drastic reduction in the number of cases of illness in developed countries^4^. However, bronchiectasis continues to be prevalent in developing countries such as India, Turkey, and Brazil^5^.

Although many cases can be treated non-surgically^6^, surgery is an excellent therapeutic option for patients with localized bronchiectasis who remain symptomatic despite the clinical treatment optimization, with good results and low postoperative mortality^7^ ^-^ ^9^. Some selected patients with non-localized bronchiectasis may also benefit from surgical treatment^2^ ^,^ ^7^.

In recent decades, video-assisted thoracic surgery (VATS) has been recommended to treat malignant lung cancer^10^. However, there is no consensus regarding VATS in treating suppurative diseases such as bronchiectasis. Only some studies in this area show the clinical results of long-term use of this technique^9^ ^,^ ^11^ ^,^ ^12^. 

The present study aims to evaluate the early and late results of surgical treatment of patients with bronchiectasis, comparing the VATS approach versus the open thoracotomy (OT).

## METHODS

Observational retrospective study of patients who underwent surgery for bronchiectasis, between September 2005 and October 2019, in the Department of Thoracic Surgery of a major public Hospital, reference in chest diseases from the Northeast of Brazil. The data was obtained from the institutional database and complemented, if necessary, by reviewing the medical records.

The inclusion criteria were patients submitted to anatomic lung resection to treat symptomatic bronchiectasis. All of them must have the diagnosis established by high-resolution computed tomography (HRCT). The exclusion criteria were the associated diagnosis of lung abscess, aspergilloma, and Tuberculosis sequelae with extensive lung cavitation, extensive fibrosis, “lung destruction,” or bronchial stenosis. Indications for surgery included recurrent hemoptysis, recurrent pneumonia, or associated symptoms of chronic productive cough, which persisted despite adequate clinical treatment in patients whose distribution of bronchiectasis was localized or non-localized but amenable to complete resection in an extended procedure. 

Patients referred to our hospital were evaluated in our outpatient clinic, and candidates for surgery underwent preoperative laboratory tests, electrocardiograms, and routine pulmonary function tests. The HRCT evaluation in the axial, sagittal, and coronal views was obligatory to verify the distribution of bronchiectasis and to program the pulmonary resection. In addition, flexible bronchoscopy was performed to assess bleeding sites, secretion drainage, and the presence of foreign bodies or endobronchial lesions. Bronchial lavage was sent for microbiological study. In case of infection confirmed by bacterial culture, patients were treated with antibiotics preoperatively for one week and continued postoperatively. Prophylactic antibiotics were given for patients without active infection, starting on the day of surgery.

The variables collected were gender, age, preoperative symptoms, etiology of bronchiectasis, lung, lobe or segments involved, FVC and FEV1 and type of surgical resection. The early outcomes assessed were postoperative: complication, and mortality in the first 30 days after surgery or the same hospitalization, and length of hospital stay The late surgical results were classified as excellent, complete remission of the symptoms; good, significative improvement of the symptoms; and poor, little or no improvement of symptoms.

Patients were divided into two groups according to the surgical access used: Group A, patients undergoing conventional surgery by conventional Open Thoracotomy (OT), and Group B, patients operated by Video-Assisted Thoracic Surgery (VATS). The type of access was defined by the surgical team based on personal experience with the preference to perform VATS for cases with limited pleural thickness, without calcified hilar or peribronchial lymph nodes on CT as suggested by Yen et al.^12^.

The Research Ethics Committee approved the project with a registry number of 3.370.849..

### Surgical technique

All patients underwent general anesthesia and tracheal intubation with a double-lumen tube to avoid contralateral contamination by secretions and allow for one-lung ventilation. In the OT group, access to the thoracic cavity was performed by lateral thoracotomy with muscle preservation on the 5^th^ intercostal space (ICS), and in the VATS group, by bi-portal access, with a portal for the camera placed in the 8^th^ ICS in the midaxillary line and a working portal through a 4cm incision on the 5^th^ ICS in the anterior axillary line, protected by a plastic wound retractor, without rib spreading. Exceptionally, in the case of thick adhesions, a third port was added on the 8^th^ ICS in the posterior axillary line. 

In all cases, anatomical lung resection was performed, which was considered complete when all affected segments, defined preoperatively by computed tomography, were resected by lobectomy or segmentectomy in the localized disease and by combined resections such as lobectomy, lobectomy plus segmentectomy, or segmentectomy bilateral in nonlocalized and multisegmented disease. In bilateral disease, surgery was performed in two surgical procedures, with an interval of at least three months between each surgery.

For pain control, in the OT group, epidural morphine was given preemptively at the beginning of the surgery and supplemented, on the first postoperative day, with non-steroidal anti-inflammatory drugs and intravenous opioids, which on subsequent days were administered orally. In the VATS group, the only difference was that instead of epidural morphine, an intercostal block was performed with ropivacaine. In the postoperative period, in case of persistent pain, in any of the groups, supplemental morphine was administered intravenously or subcutaneously. Postoperative management included intensive respiratory physiotherapy for all patients.

Patients were followed up in our outpatient clinic by a member of the surgical team or by the reference pulmonologist, who recorded information about disease progression concerning preoperative symptom control. For patients who did not return to the outpatient clinic, one researcher kept contact by telephone to obtain clinical information.

### Statistical analysis

Data were collected in forms and stored in an SPSS STATISTICS software version 22 spreadsheet (IBM Corp, NY, USA) for analysis. Comparison of means for quantitative variables was performed using Student’s t-test or Mann Whitney U test for non-normal continuous variables, and categorical variables using the chi-square or Fisher’s exact test. Multivariate linear regression models with robust HC3 errors were performed for continuous early outcomes and logistic multivariate regression models for binary early outcomes, adjusted for patient age and sex. The P value <0.05 was considered statistically significant.

## RESULTS

The OT and VATS groups comprised 54 (52.4%) and 49 (47.6%) patients. Demographic and clinical characteristics regarding age, gender, symptoms, location of bronchiectasis, and lung function were similar (p>0.05) in both groups, as detailed in Table 1.

**Table 1:** Clinical characteristics of patients in the thoracotomy and video-assisted thoracic surgery group.

Variables	OT n=54	VATS n=49	p value
Age (years) ± SD	40.6 ± 15.0	39.8 ± 15.0	0.93
Gender (female/male)	38/16	32/17	0.58
Symptoms, n(%)			
Hemoptysis*	30 (55.5)	35 (71.4)	0.09
Chronic cough	30 (55.5)	26 (53.0)	0.8
Repeated respiratory infection	26 (48.1)	22 (44.9)	0.7
Dyspnoea	7 (13.0)	9 (18.4)	0.4
Chronic sinusitis	5 (9.2)	4 (8.2)	0.8
Chest pain	4 (7.4)	3 (6.1)	0.8
Localized/non-localized bronchiectasis	39/15	38/11	0.53
% FVC	75.04 ± 17.5	87.4 ± 25.3	0.46
% FEV1	67.6 ± 19.6	82.2 ± 19.8	0.58

SD: standard deviation; OT: open thoracotomy; VATS: video-assisted thoracic surgery; FVC: forced vital capacity; FEV1: forced expiratory volume in one second; *Massive hemoptysis in 9 (14%) cases.

All patients were symptomatic when surgery was indicated, with hemoptysis, chronic productive cough, and recurrent respiratory infection being the most common symptoms. Dyspnea, chest pain, and signs of chronic sinusitis were less common. Of the 26 patients with non-localized bronchiectasis, 10 had bilateral disease (Table 1). Regarding the etiology, tuberculosis, and pneumonia have predominated as the leading causes, with other less common ones, such as ciliary dyskinesia and foreign body. Based on tomographic findings, the initial diagnosis was lower lobe bronchiectasis in two patients with pulmonary sequestration. However, in most cases (40.8%), it was impossible to clinically establish the cause of bronchiectasis, which was considered to have an undetermined etiology (Table 2).

**Table 2:** Etiology of bronchiectasis in the thoracotomy and video-assisted thoracic surgery (VATS) group.

Etiology n (%)	Thoracotomy n=54	VATS n=49	Total n (%)	p value
Post-tuberculosis	16 (29.6)	11 (22.4)	27 (26.2)	0.408*
Post-pneumonia	13 (24.0)	11 (22.4)	24 (23.3)	0.846*
Ciliary dyskinesia	3 (5.5)	1 (2.0)	4 (3.9)	0.619**
Foreign body	1 (1.8)	2 (4.0)	3 (2.9)	0.604**
Pulmonary sequestration	1 (1.8)	1 (2.0)	2 (1.9)	1.00**
Immunoglobulin deficiency	1 (1.8)	0 (0.0)	1 (1.0)	1.00**
Undetermined	19 (35.2)	23 (47%)	42 (40.8)	0.225*

*Chi-square; **Fisher’s Exact Test.

The surgeries were performed using the techniques described in the methodology, with 108 surgical resections being performed in the 103 patients, of which 98 were unilateral, and five were bilateral in two surgical stages. Isolated lobectomy was the most common type of resection (65/103 = 63%), followed by combined resections, such as lobectomy with associated anatomical segmentectomy, or resections of lung segments from different lobes, such as lingulectomy and basilar segments (25/108 = 23%). Nine pneumonectomies were performed, eight in the OT group and one in the VATS group (Table 3). Resection was considered complete in 100% (77) of the patients with localized bronchiectasis and 81% (21 of 26 patients) of those with non-localized bronchiectasis.

**Table 3:** Surgical procedures performed in the thoracotomy and video-assisted thoracic surgery (VATS) groups..

Type of resection	Thoracotomy n (%)	VATS n (%)	Total n (%)
First surgery	54 (52.4)	49 (47.6)	103 (100)
Pneumonectomy	8 (89)	1 (11)	9 (8.7)
Bilobectomy	2 (40)	3 (60)	5 (4.8)*
Lobectomy	30 (46)	35 (54)	65 (63)
Lobectomy and segmentectomy	6 (86)	1 (14)	7 (6.8)*
Lobectomy and lingulectomy	4 (80)	1 (20)	5 (4.8)*
Lingulectomy	2 (40)	3 (60)	5 (4.8)
Pyramidectomy	0	3 (100)	3 (2.9)
Pyramidectomy and lingulectomy	2 (50)	2 (50)	4 (3.9)*
Second surgery	2 (40)	3 (60)	5 (100)
Lobectomy and lingulectomy	1 (100)	0	1 (20)*
Lobectomy and pyramidectomy	1 (50)	1 (50)	2 (40)*
Pyramidectomy and lingulectomy	0	1 (100)	1 (20)*
Lingulectomy	0	1 (100)	1 (20)

VATS: video-assisted thoracic surgery; *Combined resections at the first surgery, 21 (20.4%), and second surgery, 4 (80%).

### Early results

A high percentage of complications was observed, but no difference between the OT (29.6%) and VATS (24.5%) groups. No mortality was encountered in either group. Post-operative hospital stay was shorter in the VATS group (5,4± 3.07 days) compared to the OT group (8,7±5,8 days, p=0.029) (Table 4). Regarding complications, there was a lower tendency for complications in the VATS group (12 complications, 24.5%) versus OT (19 complications, 29.6%), but with no statistical significance, p=0.55. The most common complications in both groups were prolonged air leak (total = 5 cases, 4.8%; OT=3, VATS=2), pleural empyema (total = 4 cases, 3.9%; OT=3, VATS=1) and pneumonia (total = 4 cases, 3.9%; OT=3, VATS=1). Other less common complications: atelectasis (total = 2 cases, 1.9%; OT=1, VATS=1), pleural effusion (total = 2 cases, 1.9%; OT=0, VATS=2), bronchopleural fistula (total = 2 cases, 1.9%; OT=1, VATS=1), hemoptysis (total = 2 cases, 1.9%; OT=0, VATS=2), seroma in surgical wound (total = 2 cases, 1.9%; OT=0, VATS=2) residual pleural space (total = 1 case, 1.0%; OT=1, VATS=0), retained hemothorax (total = 1 cases, 1.0%; OT=1, VATS=0), surgical site infection (total = 1 cases, 1.0%; OT=1, VATS=0), surgical site hematoma (total = 1 cases, 1.0%; OT=1, VATS=0), surgical wound dehiscence (total = 1 cases, 1.0%; OT=1, VATS=0), pulmonary thromboembolism (total = 1 cases, 1.0%; OT=1, VATS=0), atrial fibrillation (total = 1 cases, 1.0%; OT=1, VATS=0) and supraventricular tachycardia (total = 1 cases, 1.0%; OT=1, VATS=0),

**Table 4:** Immediate surgical results in the thoracotomy and video-assisted thoracic surgery (VATS) groups.

Variables	Thoracotomy n = 54	VATS n = 49	p value
Postoperative complications	16 (29.6%)	12 (24.5%)	0.549*
Length of hospital stay (day±SD)	8.7 ± 5.8	5.4 ± 3.07	0.002*
Operative mortality (30 days)	0	0	-
Conversion to thoracotomy	-	4 (8.2%)	-

VATS = video-assisted thoracic surgery; *Multivariate model adjusted for age and gender.

In the VATS group, there were four conversions to open thoracotomy, two due to technical difficulty in releasing firm adhesions and fused fissure, one due to bleeding, and one due to inadequate selective intubation.

### Late results

Seventy-seven (75%) patients were followed up for 56.7 ± 44.3 months (median 50 months), and of these patients, the results were considered excellent in 55 (71.4%) cases, good in 20 (26%), and poor in 2 (2.6). The tendency for better late clinical results in the VATS group than in the OT group, with excellent 83.3% and 64.1% of the cases, respectively; was not statistically significant the p-value was 0.06. On the other hand, analyzing the result concerning the distribution of the bronchiectasis, the excellent percentage was 82.1% in patients with localized bronchiectasis and only 47.4% in those with non-localized bronchiectasis, p=0.003 (Table 5).

**Table 5:** RLate surgical results from the follow-up of 77 patients in the thoracotomy and video-assisted thoracic surgery (VATS) group.

	OT n=39 (%)	VATS n=36 (%)	p value
Excellent Good	25 (64.1) 14 (35.9)	30 (83.3) 6 (16.7)	0.06
	Localized bronchiectasis n=56 (%)	Non-localized bronchiectasis n=19 (%)	
Excellent Good	46 (82.1) 10 (17.9)	9 (47.4) 10 (52.6)	0.003

OT: open thoracotomy; VATS: video-assisted thoracic surgery.

## DISCUSSION

Bronchiectasis is defined as an irreversible dilation of the airways, usually associated with chronic bacterial infection and symptoms of cough and sputum^14^. It is a heterogeneous disease in terms of etiology, clinical manifestations, and radiological spectrum, with many patients without a defined etiology^14^ ^,^ ^15^. 

In the present study, the patients belonged to a young age group, mean age of 40 years, which is in line with the recent series, ranging from 30 to 50 years^5^ ^,^ ^7^ ^,^ ^15^ ^-^ ^19^. The literature describes chronic productive cough as the most common symptom, followed by hemoptysis^7^ ^,^ ^15^ ^,^ ^17^ ^,^ ^20^. Still, the present study was the opposite: hemoptysis was present in 63%, followed by productive cough in 54% of cases. This can be explained by the higher prevalence of tuberculosis etiology (26.2%) and the unknown or undetermined etiology in 40.8% of cases. However, similar findings were described by Ceylan et al.^15^. In 50 patients operated on for bronchiectasis, 20 by VATS, and 30 by thoracotomy; the most frequent symptom was hemoptysis (62%), and the etiology of bronchiectasis was defined in only 22% of cases. 

The goals of treating bronchiectasis are to reduce symptoms, prevent exacerbations, improve quality of life, and halt disease progression^22^ ^,^ ^23^. The role of surgical resection of the affected lung in the management of bronchiectasis is still being determined and sometimes controversial, depending on the service expertise and infrastructure. However, most authors agree with the indication of surgical treatment for patients with bronchiectasis located in a single lobe or segment, with symptoms not controlled by conventional clinical therapy or life-threatening complications, such as massive hemoptysis^12^ ^,^ ^23^ ^-^ ^25^.

In the present study, all patients were symptomatic and had the diagnosis of bronchiectasis made by HRCT and confirmed by anatomopathological examination in the postoperative period. Forty-nine (47.6%) underwent vats resection and 54 (52.4%) thoracotomies, of which 77 patients had localized bronchiectasis and 26 non-localized, of which 10 were bilateral. Patients were similar in both groups (VATS and OT) regarding age, gender, type of bronchiectasis, and lung function (FVC and FEV1).

Sixty-five (63%) lobectomies were performed, of which 54% were performed by video, which is in agreement with other series that performed lobectomies as the type of resection between 50 and 81%^17^ ^,^ ^18^ ^,^ ^20^ ^,^ ^26^. Although minimally invasive approaches like VATS or robotic are considered the surgical approaches of choice for lung cancer, they have not yet become routine for patients with bronchiectasis^17^ ^,^ ^18^ ^,^ ^27^. 

In the present study, no difference in surgical complications was demonstrated between the VATS vs OT groups (24.5% vs 29.6%, p=0.55), with prolonged air leak (4.8%), pleural empyema (3.9%) and pneumonia (3.9%) the most common in both groups. Other studies also found prolonged air leaks to be the most frequent complication^15^ ^,^ ^20^ ^,^ ^21^. Our length of hospital stay was shorter in the VATS group (VATS, 5.4 days vs OT, 8.7 days, p=0.029), which reflects the minimally invasive aspect of the VATS group, allowing faster recovery. In some studies that compared the results of VATS with thoracotomy in the surgical treatment of bronchiectasis, the authors also found no difference in complications between the two groups, with percentages ranging from 16.3 to 38.9% in VATS and from 18 to 43.4% in thoracotomy^15^ ^,^ ^18^ ^,^ ^20^ ^,^ ^26^. Zhang et al. in 2011, showed both a decrease in complications (15% vs 26%) and the length of hospital stay (11 days vs 14 days) in patients undergoing VATS over thoracotomy^17^. The relatively high general complications rate is compatible with other studies for this population and could be attributed to the inherent severity of the disease^29^. 

In our series, there was no surgical death in either group. The VATS group had an 8.2% conversion rate to open surgery. Recent series in the literature, both for VATS and thoracotomy surgeries, show mortality rates ranging from zero to 2.9%, and conversion rates ranging from zero to 18.7%, which are increasingly smaller as surgeons gain experience with video surgery in inflammatory diseases^7^ ^,^ ^17^ ^-^ ^20^ ^,^ ^26^. In our study, in the first seven years, of 54 surgeries performed, only 37% (20 cases) were performed by VATS , and in the last seven years, of 49 surgeries, 59% (29 cases) were performed via video, with only one conversion (3.4%).

Regarding the effectiveness of surgery for symptom improvement, there are many series of open thoracotomy surgery that evaluate this outcome. Still, few articles on video surgery for bronchiectasis evaluate late surgical results concerning symptom control^17^ ^,^ ^18^ ^,^ ^20^. Zhou et al.^18^, analyzing results of patients who remained asymptomatic with those who had improvement in symptoms, showed 92% effectiveness of surgeries performed by thoracotomy and 94.3% of surgeries performed by VATS, with no difference between the two groups.

Seventy seven patients were followed for 56.7 ± 44.3 months; the results were excellent, with complete control of symptoms in 64.1% of cases and good in 35.9% in the OT group versus 83.3% and 16.7% in the VATS group, with no difference between groups (p=0.06), with a trend towards better results for VATS, which is in line with some series in the literature^17^ ^,^ ^18^ ^,^ ^20^. In the evaluation regarding the location of the bronchiectasis, although it was not the focus of this study, significantly better results were observed in patients with localized bronchiectasis than in those with non-localized bronchiectasis, being excellent in 82.1% of cases vs 47.4% (p=0.003) respectively, which is comparable with other series^7^ ^,^ ^18^. Dai et al. (2017) showed that performing resection of the most affected areas, which they called predominant lesion, in non-localized cases and leaving small foci of disease in the ipsilateral or contralateral lung, the result was significant symptomatic improvement, considered excellent in 62.2% of patients and good in 27%^30^.

The present study has some limitations. First, the retrospective nature of it. Second, the allocation criteria of patients in the groups were not done by randomization or propensity score. However, the patients in the two groups are similar in some aspects, such as age, sex, location of bronchiectasis, and lung function. Third, due to the lost follow-up, only 75% of the patients could be evaluated for late clinical outcomes. Selection bias could also be present in the choice of surgical approach, considering that the VATS group was preferentially chosen among patients with less pleural thickening, without calcification of hilar lymph nodes, or by the surgeon’s judgment. These factors may have had some influence on the postoperative results. Lastly, the statistical groups for the comparative evaluation are small. Therefore, the results should be interpreted with great caution.

## CONCLUSIONS

We concluded that the morbidity and mortality of patients with bronchiectasis operated on using the video-assisted technique were similar to those operated on through thoracotomy. However, those using the video technique had shorter hospital stays reflecting the early recovery. Late results were excellent in most patients, being better in patients with localized bronchiectasis.
